# The role of neuronal nitric oxide and its pathways in the protection and recovery from neurotoxin-induced *de novo* hypokinetic motor behaviors in the embryonic zebrafish (*Danio rerio*)

**DOI:** 10.3934/Neuroscience.2019.1.25

**Published:** 2019-03-28

**Authors:** Amber Woodard, Brandon Barbery, Reid Wilkinson, Jonathan Strozyk, Mathew Milner, Patrick Doucette, Jarred Doran, Kendra Appleby, Henry Atwill, Wade E. Bell, James E. Turner

**Affiliations:** Department of Biology, Center for Molecular, Cellular, and Biological Chemistry, Virginia Military Institute, Lexington, VA 24450, USA

**Keywords:** neuronal nitric oxide synthase (nNOS), nNOS inhibitor, DTT, ODQ, motor dysfunction, dopamine, 6-hydroxydopamine neuronal toxicity, zebrafish

## Abstract

Neuronal nitric oxide (nNO) has been shown to affect motor function in the brain. Specifically, nNO acts in part through regulation of dopamine (DA) release, transporter function, and the elicitation of neuroprotection/neurodegeneration of neurons in conditions such as Parkinson's disease (PD). Recently, the zebrafish has been proposed to be a new model for the study of PD since neurotoxin damage to their nigrostriatal-like neurons exhibit PD-like motor dysfunctions similar to those of mammalian models and human patients. Results from this study demonstrate that treatment of 5 days post fertilization (dpf) fish with a nNO synthase inhibitor as a co-treatment with 6-OHDA facilitates long-term survival and accelerates the recovery from 6-OHDA-induced hypokinesia-like symptoms. These findings are unique in that under conditions of neurotoxin-induced stress, the inhibition of the NO-related S-nitrosylation indirect pathway dramatically facilitates recovery from 6-OHDA treatment but inhibition of the NO-sGC-cGMP direct pathway is essential for survival in 5 dpf treated fish. In conclusion, these results indicate that nNOS and the inhibition of the NO-linked S-nitrosylation pathway plays an important role in antagonizing the protection and recovery of fish from neurotoxin treatment. These data begin to help in the understanding of the role of NO as a neuroprotectant in dopaminergic pathways, particularly those that influence motor dysfunctions.

## 1. Introduction

Movement disorders are prominent symptoms of a number of neurodegenerative diseases such as Parkinson's (PD) and Alzheimer's disorders. For example, PD causes the systematic degeneration of dopamine (DA) neurons in the basal ganglia (BG) of the brain [1]. According to the Mayo Clinic, those with this movement disorder exhibit tremors, bradykinesia (hypokinesia), rigidity, balance and posture impairment, loss of automatic movements, and speech difficulties [2]. PD affects millions across the world; the European Parkinson's Disease Association states that 6.3 million people have the neurodegenerative disorder globally [3]. Those who suffer with PD are without a cure, and must resort to methods of PD treatment for relief. Currently, the “gold standard” treatment for PD is the use of levodopa (L-DOPA).

Several signaling molecules have been implicated in the neuromodulation/neuroprotection of DA neurons in the nigorstrital (BG) pathway associated with either animal models of MPTP (1-methyl-4phenyl-1,2,3,6-tetrahydropyridine) or 6-OHDA (2,4,5-trihydroxyphenethylamine) neurotoxicity that create PD-like symptoms, or from PD patient clinical data. One of these signaling molecules is nitric oxide (NO). NO, a gas released by the actions of the NO synthase (NOS) enzyme on L-arginine, acts as a signaling molecule with direct actions on existing metabolic pathways, as well as through genomic mechanisms [4],[5]. By virtue of its gaseous state, NO can diffuse across cellular membranes without the aid of membrane bound transport proteins or receptors and can interact directly with its end targets either in the cell in which it was synthesized or in surrounding cells. In turn, its actions are precisely controlled due to its very short half-life and restricted diffusion distance [4],[5]. At higher concentrations NO can act as a free radical in some situations or it may bind to superoxide anion (O_2_^−.^), causing pathophysiological effects [6]. Specifically, owing to very unstable nature, NO gets converted into nitrite, peroxynitrite and other reactive nitrogen species that could lead to nitrosative stress in the nigrostriatal system. Nitrosative stress is widely implicated in Parkinson's disease (PD), and its beneficial and harmful effects are demonstrated in *in vitro*, rodent and primate models of toxins-induced parkinsonism and in the blood, cerebrospinal fluid and nigrostriatal tissues of sporadic PD patients [7]. It is under these conditions that NO is thought to play a role in the genesis of such neurological diseases as PD [8]. On the other hand, NO at lower concentrations can act as a cellular protectant through prevention of apoptosis, excitotoxicity, neuronal depolarization, and regulation of the redox state in the mitochondria [9],[10]. NO acting at the cellular level interacts with either its soluble guanylyl cyclase (sGC) receptor molecule to produce cyclic guanosine monophospahte (cGMP) which activates a cascade of cellular enzymes or causes *S*-nitrosylation of cysteine residues leading to protein conformational changes [11],[12]. These two pathways are called NO-sGC-cGMP-dependent or NO-sGC-cGMP-independent pathways respectively. In the BG one of the four nitric oxide synthase (NOS) isoforms, neuronal nitric oxide synthase (nNOS), is believed to act through the NO-sGC-cGMP-dependent pathway that serves to modulate transcription factors, phosphodiesterases, ion-gated channels, or cGMP-dependent protein kinases (PKG), each of which continues to act physiologically in the nervous system [13]. In the BG, NO has been shown to affect DA release, influence transporter function, and elicit neuroprotection of DA neurons [14].

A second signaling molecule that has been implicated in the neuromodulation/neuroprotection of DA neurons in the nigorstrital (BG) pathway is estrogen (E2). Previous studies have suggested that E2 has neuroprotective effects in DA neurons and can regulate the synthesis of DA as a pro-dopaminergic agent [15]. In addition, studies show that DA neurons of the central nervous system have E2 receptors and the presence of the E2 synthesis enzyme aromatase [15]. It is clear that there is a connection between E2, the central nervous system, and movement disorders like PD. Indeed, pre-menopausal women are less likely to show PD symptoms with a majority of patients being male and over 60 [16]. Thus, there appears to be a sexual dimorphism between males and females when it comes to PD prevalence [16]. As a result of the hormonal differences, E2 is considered a neuroprotectant molecule, but there is no evidence for a similar role for testosterone [16]. Recently, this effect has been examined in female rats who have been treated with the MPTP neurotoxin and have shown the ability to resist muscular activity loss compared to males [16]. In addition to being neuroprotective, there is also accumulating evidence that E2 may also cause detrimental effects such as hyperkinetic/chorea/dystonia symptoms in females on post-menopausal replacement therapy after hysterectomy [15]. There is also the recent case of a patient suffering from adult onset Sydenham's chorea who discontinued E2 replacement therapy and months later these hyperkinetic/chorea symptoms were significantly diminished [17]. Part of the mechanism by which E2 may exert its influence in the BG of PD patients is through its documented influence on nitric oxide (NO) levels through its regulation of the expression of nitric oxide synthase (NOS) [18].

Zebrafish have been found to be an excellent model for studying motor disorders because they show similar neurological functions that humans possess and can easily demonstrate PD-like symptoms with damage to its basal ganglia-like structures [19]. In turn, a model where BG-like pathways are simpler and the DA neurons fewer in number and easier to visualize and access would be ideal for such studies. The embryonic zebrafish would appear to fit these criteria. The DA system has been well characterized in both embryo development and in adult fish. The DA system in zebrafish, which is equivalent to the nigrostriatal pathway in mammals, has been shown to ascend to the subpallium (striatum) from the basal diencephalon [20]. Also, zebrafish embryos and adults respond to the DA neurotoxins MPTP and 6-OHDA, as well as to the DA receptor agonists/antagonists in much the same manner as in mammalian models of PD [21],[22]. Indeed, there are an increasing number of studies which make a case for the use of zebrafish as a model for the study of movement disorders such as PD [19]. Earlier observations from our lab have established a zebrafish locomotor dysfunction model linked to both E2 and NO deficiency [23]–[27].

It is the hypothesis of this study that embryonic zebrafish treated with the neurotoxin 6-OHDA will be rescued by modulation of NO and E2 levels through co-treatment with nNOSI or AI. The main goal of this study is to demonstrate that rescue and recovery effects from 6-OHDA treatments that are proposed to be linked to the NO-sGC-cGMP-dependent pathway. Conversely, inhibition of the NO-sGC-cGMP-independent pathway (S-ntrosylation) will protect neurotoxin treated fish from the hypokinetic phenotype. Our current data confirms this hypothesis. These findings begin to help in the understanding of the role of NO and its pathways as a neuroprotectant in dopaminergic pathways, particularly those that influence motor dysfunctions.

## 2. Materials and methods

### 2.1. Fish preparations

The compound *roy;nacre* double homozygous mutant zebrafish, named *casper*, were used in this study. Casper shows the effect of combined melanocyte and iridophore loss in which the body of the embryonic and adult fish is largely transparent due to loss of light absorption and reflection. These transgenic fish were obtained from Carolina Biological Supply. All fish were maintained in a basic embryonic rearing solution (ERS) consisting of NaCl, CaCl_2_, KCl, and MgSO_4_. These necessary ions were dissolved in deionized water containing a 0.05% methylene blue solution, which served as an antimicrobial agent. All solutions were changed every 24 hours and embryos were incubated at 28 °C. All reagents were obtained from Sigma-Aldrich, and solutions were made daily before use unless noted otherwise. Fish treated at 4-6 days post fertilization (dpf) were allowed to hatch on their own prior to treatment. This particular later stage of fish development was chosen for this study over our earlier stage studies due to a greater response to various treatments. Specifically, past research from our lab established a model based on exposing the zebrafish aged two days post fertilization (dpf) to whatever specific experimental treatment was being observed for a four-day time period [25]. However, this model often yielded a low but significant percentage of phenotypical responses. To maximize the phenotypic response, this current study focused on creating a model that would yield maximum phenotypic changes under a shorter duration. The current zebrafish model focuses on treating fish four to six dpf as opposed to two dpf. This four to six dpf model was more successful than the two dpf model in generating significant and consistent phenotypic results in the zebrafish. All procedures were in accordance with NIH guidelines for the care and treatment of animals.

### 2.2. Reagents

Embryonic zebrafish were administered their prescribed reagents through absorption from their incubation medium. The optimal concentrations for reagents used in this study are based on previously published dose response studies [23]–[27]. When two reagents are given together in the same treatment, they are referred to as co-treatments. The removal of a reagent from the solution by another if referred to as a washout.

#### 2.2.1. NO related reagents

All NO-related reagents for treating zebrafish have been previously tested in a dose response paradigm to insure optimal results and proper survival. Baseline target concentrations were identified based on previously published data. Proadifen hydrochloride (Sigma # P1061) was used as a selective nNOS inhibitor (nNOSI). Our previous studies have shown that nNOSI was more effective in our model system than the other two inhibitors, eNOSI and iNOSI [23]–[27]. With ERS as the diluent, a 50 µM concentration provided optimal results in its production of creating the listless condition and this dose was used throughout the current study.

Diethylenetriamine/nitric oxide adduct (DETA-NO, Sigma) was used to provide a slow extended release of exogenous NO as a co-treatment with some of the inhibitors used in the experiments in an effort to show that NO inhibition mediated symptoms exhibited by fish can be rescued. It was dissolved into ERS resulting in a 50 µM concentration which provided the best results.

1H-[1,2,4] Oxadiazolo [4,3-a] quinoxalin-1-one (ODQ, Sigma) was used as a soluble guanylyl cyclase (sGC) inhibitor which compromises the NO-sGC-cGMP-dependent pathway by reducing cGMP production. It was dissolved into a 0.1% DMSO solution then diluted with ERS to a working concentration of 30 µM for application. In addition, DTT (dithiothreitol, Sigma) was used as an inhibitor of the NO-sGC-cGMP-independent pathway which prevents S-nitrosylation events at a concentration of 100 µM.

#### 2.2.2. DA related reagents

The neurotoxin 6-hydroxy-dopamine (6-OHDA, Sigma) was used at a concentration of 250–500 µM to illicit motor deficits by damaging the DA neurons in the equivalent of the zebrafish nigrostriatal-like pathway as established previously in the literature [27].

#### 2.2.3. E2 related reagents

All E2-related reagents for treating zebrafish have been previously tested in a dose response paradigm to insure optimal results and proper survival [26]. Based on previous studies, E2 (17*β*-Estradiol, Sigma) used at 1, and 5 µM, as established previously and initially solubilized in a 100% ethanol stock solution diluted down to the base treatment solution with ERS, ensuring that the ethanol concentration in the final solution was equal to or lower than 0.5%. The control group will consisted of ERS salt solution plus 0.05% ethanol. The AI (4-androstene-3, 17-dione,4-OH-A, MW-286.4, Sigma #A9630) was used as an aromatase inhibitor (AI) to block the production of E2 from androgens [24],[25]. It will be used at 50 µM and made from a 100% ethanol stock solution diluted down to the base treatment solution with ERS, ensuring that the ethanol concentration in the final solution was equal to or lower than 0.5%.

### 2.3. Experimental design

#### 2.3.1. Staged activities of fish in response to 6-OHDA treatment

**Figure 1.**
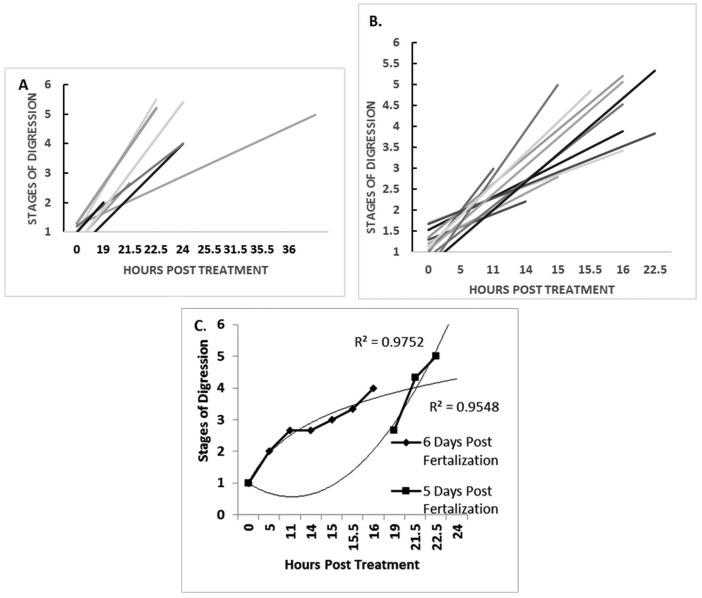
Graphs comparing the stages of digression (see Table 1) in 5 and 6 dpf fish in response to 6-OHDA treatment. A–B: A demonstration of the trend lines of linear relationships between stages of digression and time of passage through the various stages of digression for twelve separate fish over a 22–36 hour period of treatment. C: A linear regression analysis comparing the rates of progression through the various regression stages (1–5) leading towards the listless phenotype (stage 5) in 5 compared to 6 dpf 6-OHDA treated fish.

This study will look at the staged activities of each of twelve 5 and 6 dpf fish in response to 6-OHDA over 22–36 hours of treatment (see Figure 1). A linear relationship will be established for each fish by comparing time after treatment with the stages of digression that they will pass through (see Table 1). This analysis will indicate whether all fish follow the same chronological order of digression within a tight timeframe that is completed within the first 24 hours of treatment. In particular, the slopes of the lines for each fish will indicate if developmental age determines the scale of response to 6-OHDA treatment. In particular, a linear regression analysis will be undertaken to compare the rates of progression through the various regression stages (1–5) leading towards the listless phenotype (stage 5) in 5 compared to 6 dpf 6-OHDA treated fish (see Table 1, see Figure 1C).

#### 2.3.2. Effects of E2 and 6-OHDA o swimming activity

**Figure 2.**
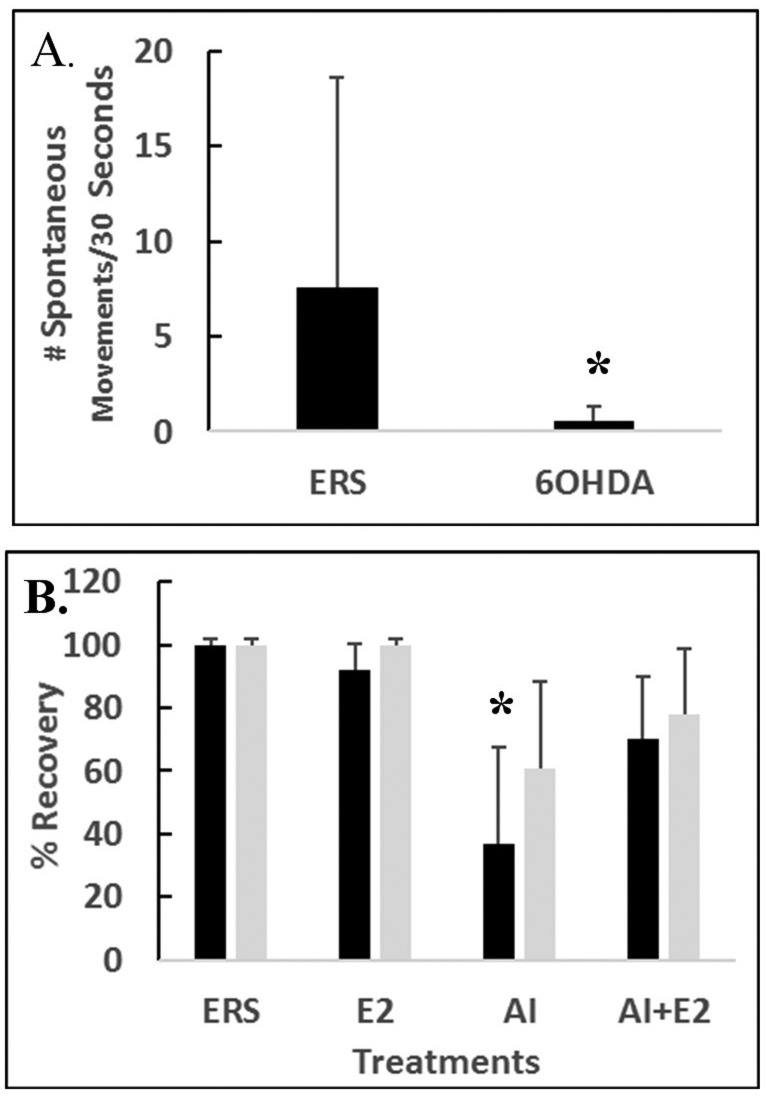
Swimming behaviors of 5dpf treated fish with either ERS, 6-OHDA, E2, or AI. A: Fish treated with ERS or 6-OHDA and tested for the number of spontaneous movements/30 seconds at 3 hours after exposure. B: Percent of fish showing the listless phenotype after 3 hours under various treatments (black bars). Light bars represent percent of recovery from the listless phenotype observed at 6 hours after an ERS washout. Error bars represent ± SD. Asterisk = *p* < 0.05.

This study will analyze the effects of E2 and 6-OHDA on the swimming behavior of 5 dpf fish after 3 hrs under various treatments. Specifically, fish sill be treated with ERS or 6-OHDA and then tested for the number of spontaneous movements/30 seconds at 9 hours after exposure (see Figure 2A). In addition, percent recovery from the listless phenotype will be recorded after 9 hours of exposure to either ERS, 6-OHDA, E2, or AI with additional data collected 3 hours after ERS washout (see Figure 2B).

#### 2.3.3. The effects of various NO treatments on survival and swimming behavior on 6-OHDA treated fish

**Figure 3.**
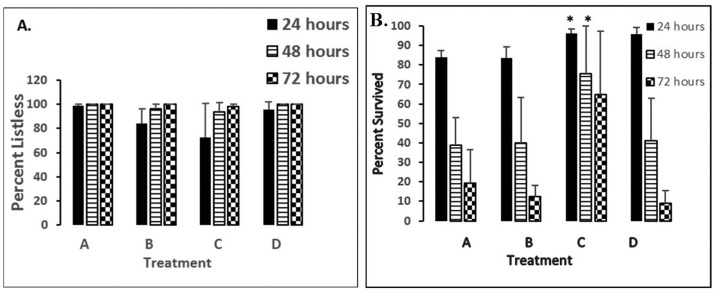
Shows percentages of 5 dpf demonstrating either listless (A.) or survival (B.) phenotypes under various treatment conditions after 24–72 hours of exposure. Error bars represent ± SD. Lettered groups of bars represents the following treatments: A—50 µM nNOSI; B—500 µM 6-OHDA; C—6-OHDA + nNOSI co-treatment; D—6-OHDA + DETA-NO co-treatment. Asterisk = *p* < 0.01 between groups.

This study will show percentages of 5 dpf fish demonstrating either listless or the survival phenotypes after 24–72 hours of exposure to either 50 µM nNOSI, 500 µM 6-OHDA, 6-OHDA + nNOSI co-treatment, or 6-OHDA + DETA-NO co-treatment (see Figure 3).

#### 2.3.4. Prominent characteristics leading to the listless phenotype in fish treated with and their ability to recover in response to various NO-related post-treatments

**Figure 4.**
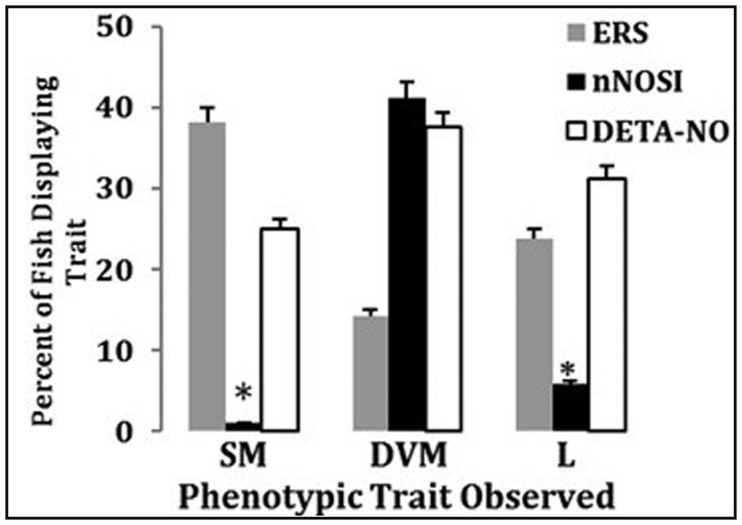
A comparison of prominent characteristics seen within the last three prominent stages (Stages 4–5) of regression (see Table 1) leading to the listless phenotype in 5 dpf as a result of fish treated with 6-OHDA for 24 hours and their ability to recover in response to various 8 hour post-treatments (washout) with either ERS, nNOSI, or DETA-NO. Error bars represent ± SD. Lettered groups of bars represents the following phenotypes: SM—spastic movements; DVF—decreased vestibular function; L—listless (hypokinetic). Asterisks = *p* < 0.001 between groups.

In this study a comparison will be made of prominent characteristics seen within the last two stages (Stages 4–5) of regression (see Table 1) leading to the listless phenotype in 5 dpf. Analyses will be made of fish treated with 6-OHDA for 24 hours and their ability to recover in response to various 8 hour post-treatments (washouts) with either ERS, nNOSI, or DETA-NO (see Figure 4). Specifically, the following phenotypes will be analyzed: SM—spastic movements; DVF—decreased vestibular function; L—listless (hypokinetic).

#### 2.3.5. Effects of prolonged nNOSI + 6-OHDA co-treatments prior to ERS washout on recovery from the hypokinetic (listless) phenotype

**Figure 5.**
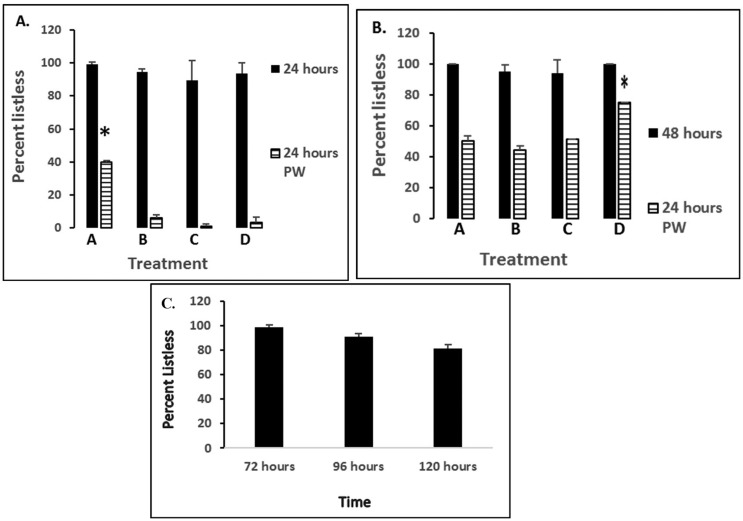
A–B: Demonstration of the effect on 5 dpf fish of prolonged 24–72 hour co-treatments with nNOSI + 6-OHDA (black bars). Light bars represent recovery from the hypokinetic (listless) phenotype after a 24-hour ERS washout. C: Results showing the percent listless phenotype after a 24-hour ERS washout in groups prior treated for 72–120 hours with nNOSI + 6-OHDA. Error bars represent ± SD. PW = time post washout. W = washout. Lettered groups of bars represents the following treatments: A—50 µM nNOSI; B—500 µM 6-OHDA; C—6-OHDA + nNOSI co-treatment; D—6-OHDA + DETA-NO co-treatment. Asterisks = *p* < 0.001 between groups.

This study focuses on the effect on 5 dpf fish of prolonged nNOSI + 6-OHDA co-treatments prior to ERS washout on recovery from the hypokinetic (listless) phenotype (see Figure 5). Specifically, 5 dpf fish will be exposed to 24–120 hour co-treatments with nNOSI + 6-OHDA. Subsequently, percent recovery from the hypokinetic (listless) phenotype will be analyzed after a 24-hour ERS washout.

#### 2.3.6. Importance of NO pathways in the recovery of 6-OHDA treated fish from post-treatment washouts

**Figure 6.**
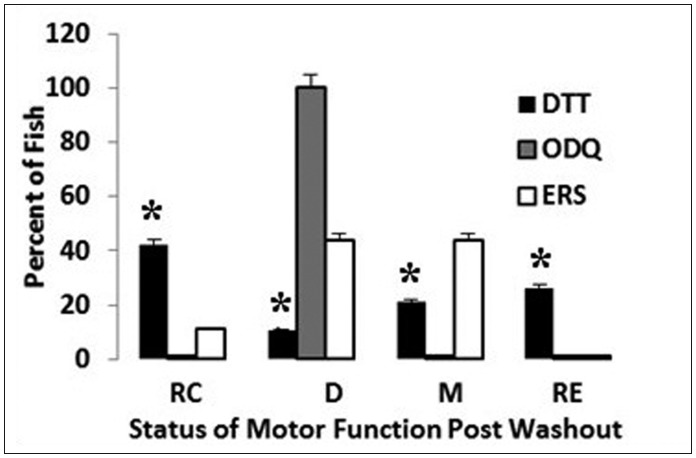
Graph depicting the importance of the two NO pathways in the recovery of 5 dpf fish from data collected at 6.5 hours of post-treatment washouts (ERS, ODQ, or DTT) preceded by a 16 hour neurotoxin treatment with 6-OHDA. Error bars represent ± SD. Lettered groups of bars represents the following phenotypes: RC—recovering; D—declining; M—maintained; R—recovered. Asterisks = *p* < 0.001 between groups.

This study undertakes to demonstrate the importance of the two NO pathways in the recovery of 5 dpf fish from post-treatment washouts following a neurotoxin treatment with 6-OHDA (see Figure 6). Specifically, 5 dpf fish will be treated with 6-OHDA for 16 hours then followed with 6.5 hours of post-treatment washouts with either ERS, ODQ, or DTT. Data relating to percent motor function recovery will be expressed in analysis of the four prominent phenotypes (recovering, declining, maintained, and recovered).

### 2.4. Data collection

For visual analysis, fish were characterized using a dissecting microscope as expressing either normal or hypokinetic dyskinesia phenotype when their swimming behaviors became significantly different from ERS controls. Specifically, the hypokinetic (listless) phenotype was identified as percent of fish found to lay on their sides and could not right themselves, were motionless and could not be induced to swim when touched by a probe on the tail as described previously in our laboratory [20]–[22]. Digression stages 1–6 were characterized as follows: 1) active, responsive, normal motor function; 2) delayed reaction time, unusual swimming pattern; 3) disorientation, decreased levels of activity; 4) spastic, erratic movements, decline in vestibular function, heart arrhythmias; 5) hypokinetic (listless) state; and 6) death. The percent survival under each experimental condition was also determined.

### 2.5. Data analysis

Data were analyzed for significant differences either by using a z-test for two population proportions or for multiple proportions using chi-square contingency table test, followed by a Marascuilo's post hoc analysis. Fish swimming movements were analyzed by a t-test or ANOVA one-way test. If the ANOVA analysis indicated a significance difference then pair-wise post hoc Tukey HSD, Scheffé, Bonferroni and Holm multiple comparisons were made of all treatments to determine significant differences between the various treatment groups. Sample sizes for all separately treated fish groups was an n = 30 and all experiments were repeated in triplicate. The only exception was the use of twelve fish in the first study (see Figure 1).

## 3. Results

### 3.1. Staged hypokinetic behaviors in response to 6-OHDA and AI treatments

Phenotypes were sorted into a six stage matrix which are referred to as the phenotypic stages of decline of zebrafish treated with 6-OHDA [Table 1]. Specifically, these phenotypes were observed in the same chronological order in all fish. Usually the first indications of 6-OHDA effects on the fish is that they show a delayed reaction time to a stimulus accompanied by unusual swimming behaviors (Stage 2) followed by further decrease in activity (Stage 3). Next, a progressive loss of vestibular function (Stage 4) is followed by the last stage which is a hypokinetc (listless) phenotype (Stage 5) prior to death (Stage 6).

**Table 1.** The six stages of phenotypic decline in 5 dpf zebrafish post 6-OHDA treatment.

Stage number	Phenotype observed
1	Active, responsive, normal motor function
2	Delayed reaction time (DR), unusual swimming pattern (USP)
3	Disorientation or decreased levels of activity
4	Spastic, erratic movements (possibly in response to stimuli); temporarily increased levels of activity, decline in vestibular function, heart arrhythmias
5	Decreased motor function descending into a hypokinetic (listless) phenotypic state
6	Death

Figure 1 A–B depicts the staged activities of each of twelve 5 and 6 dpf fish in response to 6-OHDA treatment. The linear relationship established for each fish by comparing time after treatment with the stage that they will pass through indicated that they all follow the same chronological order of digression within a tight time frame that is completed within the first 24 hours of treatment. In particular, the slopes of the lines for each fish would strongly indicate that developmental age determines the scale of response to 6-OHDA treatment. Data in Figure 1C confirms that the time line for passing through the various stages of digression is related to developmental age. In particular, a linear regression analysis was undertaken to compare the rates of progression through the various regression stages (1–5) leading towards the listless phenotype (stage 5) in 5 compared to 6 dpf 6-OHDA treated fish. Note that the high correlation coefficient values (0.9752 vs 0.9548) indicate a direct correlation between developmental age and the rates through fish pass through the staged digression phenotypes (see Table 1). Specifically, 5 dpf fish when treated with 6-OHDA developed the phenotypes of staged decline post treatment significantly slower than fish treated 6 days post fertilization. However, fish treated 5 days post fertilization moved more quickly through stages of decline once the digression began.

Figure 2A shows swimming behavior of 5 dpf fish after 3 hrs of either ERS or 6-OHDA treatments. The data shows that 6-OHDA treatment significantly diminishes the swimming behavior (*p* < 0.05). Figure 2B indicates that E2 replacement therapy can significantly (*p* < 0.05) restore recovery from AI treatment. Specifically, fish were first treated with either ERS, E2, AI, or the AI + E2 co-treatment. Data was collected at 3 hours and a second set of data collected at 6 hours of washout with ERS. The ERS control group showed 100% normal swimming behavior along with 100% after washout. E2 treated fish exhibited 92% normal swimming behavior at 3 hrs and experienced 100% recovery via ERS washout. In contrast, fish treated with AI showed only 37% normal swimming behavior at 3 hours of treatment with many fish exhibiting the listless phenotype. Additionally, the AI treated fish showed significant (*p* < 0.05) recovery after washout, 61% compared to the ERS control fish at 100%. The co-treatment (AI + E2) fish showed a significant 70% normal swimming behavior when compared to only 37% for AI only treatment group (*p* < 0.05).

### 3.2. Effects of nNOSI as a co-treatment in survival and recovery from 6-OHDA treatment

Figure 3A shows that over a 72 hour treatment period all treated groups demonstrated the listless phenotype and were not significantly different from one another (*p* > 0.05). On the other hand, Figure 3B indicates that the co-treatment (6-OHDA + nNOSI), although unable to sustain the normal swimming phenotype seen in ERS controls, was able to significantly sustain fish survival over the first 72 hours of treatment when compared to that of individual nNOSI and 6-OHDA treatments (*p* < 0.001). Specifically, the co-treated fish survival rate remained steady at 60–70% while those treated with either nNOSI or 6-OHDA alone progressively declined to a 10–20% survival rate by 72 hours post treatment. In addition, co-treatment with DETA-NO had no significant effect on sustaining fish survival (*p* > 0.05).

Figure 4 displays a comparison of prominent characteristics seen within the last two prominent stages (Stages 4–5) of regression (see Table 1) leading to the listless phenotype in 5 dpf fish treated with 6-OHDA for 24 hours and their ability to recover in response to various post-treatments (washout) with either ERS, nNOSI, or DETA-NO. When the phenotypic expressions were analyzed at 8 hours post-washout, the nNOSI post-treatment facilitated a significant (*p* < 0.001) and more rapid recovery from two of the three chosen phenotypic deficits than either ERS or DETA-NO. Specifically, significant recovery was noted in fish with abnormal motor function, spastic movements, and the listless phenotype. However, recovery from vestibular dysfunction was not better when compared to either ERS or DETA-NO poet-treatments during this short observation period. The three phenotypic expressions: SM—spastic movements; DVF—decreased vestibular function; L—listless (hypokinetic) were derived from the establishment of our behavioral model [23]–[27].

### 3.3. The ability of fish to recover from an nNOSI + 6-OHDA co-treatment

Figures 5 demonstrates the effect on 5 dpf fish of prolonged co-treatment (nNOSI + 6-OHDA) prior to ERS washout on recovery from the hypokinetic (listless) phenotype. Note that the longer the treatment prior to washout the longer it takes the fish to recover. Specifically, washout at 24 hours post treatment with ERS elicited a complete recovery from the listless phenotype 24 hours later. However, if co-treatment lasted for 48 hours prior to washout the recovery, although significant (*p < 0.0001), was much slower and not complete 48 hours later with still 40% of the population were still listless. In contrast, with co-treatment up to 72 hours and beyond fish showed no significant recovery (*p* > 0.05) from the listless phenotype after ERS post-treatment (washout).

### 3.4. The role of NO pathways in the recovery from 6-OHDA treatment

Figure 6 depicts the importance of the two NO pathways in the recovery of 5 dpf fish from post-treatment washouts following a 16 hour neurotoxin treatment with 6-OHDA. Fish post-treated for 6.5 hours with ODQ failed to survive. On the other hand, post-treatment with DTT, the inhibitor of the S-nitrosylation pathway, significantly (*p < 0.01) enhanced recovery from neurotoxin stress compared to ERS controls. Specifically, after 6.5 hours approximately 70% of fish co-treated with DTT were either fully recovered, or recovering motor activities compared to only 10% of ERS controls. None of the ODQ fish recovered but were in declining stages leading to eventual mortality.

## 4. Discussion

NO has been shown to affect motor function at several different levels [24]. Specifically, NO acts in part through regulation of DA release, transporter function, and the elicitation of neuroprotection/neurodegeneration of neurons in conditions such as PD. Recently, zebrafish have been proposed as a good model for the study of PD-like motor disorders since neurotoxin damage to their nigrostriatal-like neurons exhibit PD-like motor dysfunctions similar to those of mammalian models and human patients [19].

Results from this study demonstrate that fish at 5 dpf exhibit specific and rapid onset of staged hypokinetic behaviors through the first 24 hours of 6-OHDA treatment. These findings are in agreement with studies that have reported the effects 6-OHDA neurotoxin treatment in both early larval (2 dpf) and adult zebrafish. However, McKinley et al. [21] reported that the neurotoxin MPTP administered to 1 dpf zebrafish caused them to become completely lethargic and immobile over 3 days of treatment, in contrast to less than one day in 5 dpf as reported in the current study. Therefore, in contrasting these two studies and others including our previous work, one could conclude that there is a direct relationship between the stage of zebrafish development and the response time to a number of reagents. For example, two sets of data from the current study indicates that 5 and 6 dpf fish respond to 6-OHDA treatment on a different time line. Specifically, 5 dpf fish when treated with 6-OHDA developed the phenotypes of staged decline post treatment significantly slower than fish treated 6 days post fertilization. However, fish treated 5 days post fertilization moved more quickly through stages of decline once the digression began. In addition, current results also show that by decreasing E2 synthesis by AI treatment in 5 dpf fish exhibited the hypokinetic phenotype in less than 6 hours post- treatment compared to 3 days for 2 dpf fish [19]–[21]. A possible mechanism for this findings would be that AI decreases the synthesis of NO through is known action on the enhanced expression of nNOSI [18]. Similar age-related results were seen in fish treated with either nNOSI or L-dopa/nonoamine oxidase inhibitor treatments [26]. These results are most likely explained by the time required between early and late development periods for these various response systems to fully mature as suggested in other studies [8].

Current findings also indicated that nNOSI co-treatment of 6-OHDA fish initiates a rescue or protection phenomenon. In contrast, fish treated with just nNOSI or 6-OHDA alone do not survive well over periods beyond 24 hours. Specifically, there is only between a 10–20% survival rate in these single treatment groups at 72 hours in contrast to approximately 70% in the nNOSI + 6-OHDA co-treatment group. Adding DETA-NO to 6-OHDA as a co-treatment has no rescue value. In addition, 6-OHDA treatment of 5 dpf fish with nNOSI as a post-treatment accelerated the recovery from 6-OHDA-induced hypokinesia-like symptoms. Other studies have also reported the neuroprotective/rescue effects of nNOSI under varying conditions. Specifically, it was found that nNOSI significantly decreased L-dopa-induced dyskinesias in rats treated with 6-OHDA [29]. A possible explanation for this protective/rescue phenomenon is that 6-OHDA as a neurotoxic stressor can increase levels of NO to a point when it perpetuates and enhances the toxic environment in the fish. Similar findings by Gupta et al. [7] demonstrated that 6-OHDA was shown to increase inducible NOS expression in cultured astrocytes. Adding nNOSI to 6-OHDA provides the opportunity to decrease NO levels to such an extent that the environment is less toxic and fish naturally would survive longer. This explanation is in line with the conclusions of Padovan-Neto et al. [29] who reported that in 6-OHDA treated rats L-dopa-induced a dyskinesia accompanied by an increase in nNOS expression. However, the addition of nNOSI elicited an anti-dyskinetic effect in the rats. Several other studies have also shown that fish can be rescued from 6-OHDA treatment by co-treatments with other agents. For example, Feng et al. [30] demonstrated that both vitamin E and Sinemet (levodopa) rescued zebrafish from abnormal swimming behaviors. In addition, N-methyl-D-aspartic acid (NMDA) neurotoxicity was prevented in both mouse and gerbil brains by the addition of a NOS inhibitor [31],[32]. This line of reasoning is further suggested by the fact that at higher concentrations NO can act as a free radical in some situations or binds to superoxide anion (O_2_^−.^), causing pathophysiological effects that act to harm the body. It is under these conditions that NO is thought to play a role in the genesis of motor disorders such as PD [29]. Although nNOSI co-treated fish survived longer, they still exhibited the hypokinetic phenotype. In conjunction with this observation, NO synthesis inhibitors (NOSI) have also been shown to decrease spontaneous motor activity in various animal models, as well as the initiation of catalepsy, which is characterized by suspension of sensation, muscle rigidity, and fixity of posture [33]. Accompanying these observations, the current study also reports that there is a direct correlation between longer exposure to co-treatment (nNOSI + 6-OHDA) and the inability of fish to recover from the hypokinetic phenotype by 72–96 hours post treatment. These two observations may be explained by the fact that exposure of zebrafish to 6-OHDA treatment results in reduced motor behavior by partially or completely eliminating dopaminergic neurons in the basal diencephalon DA system [28],[30],[34]

The results from the current study also indicate that under conditions of neurotoxin-induced stress, the inhibition of the NO-related S-nitrosylation indirect pathway dramatically facilitates recovery from 6-OHDA treatment but inhibition of the NO-sGC-cGMP direct pathway is essential for survival in 5 dpf treated fish. These results could be explained by the fact that NO-induced S-nitrosylation events have been reported to facilitate neuronal cell death by inhibiting neuroprotective pathways due to its role in protein folding phenomenon [35]. In addition, it has been shown that the NO-sGC-cGMP direct pathway is essential for survival and recovery of alcohol toxicity-induced neurotoxin stress [34]. In addition, there is ample evidence accumulating in the literature that in some instances both of these pathways take part in either neuroprotective or neurodegenerative NO-stimulated events that are in agreement with the current findings [35]. Specifically, S-nitrosylation of the antioxidant enzyme peroxiredoxin 2, inhibits peroxidase activity causing the accumulation of peroxides and leads to neuronal apoptosis [30]. In contrast, it has been shown that trophic factor-deprived spinal cord motor neurons can be protected through the NO/sGC/cGMP-dependent pathway [36].

## 5. Conclusions

In conclusion, the current results from this study indicate that nNOS plays an important role in protection and recovery of fish from neurotoxin treatment. In addition, the NO-sGC-cGMP direct pathway is essential for survival and recovery of fish from neurotoxin stress. Conversely, the NO-related S-nitrosylation indirect pathway has a negative effect on recovery from 6-OHDA treatment. Specifically, when this pathway is inhibited, fish show a remarkable and robust recovery. These data begin to help in the understanding of the role of NO as a neuroprotectant/survival factor in dopaminergic pathways, particularly those that influence motor dysfunctions.
